# Genetic interrogation for sequence and copy number variants in systemic lupus erythematosus

**DOI:** 10.3389/fgene.2024.1341272

**Published:** 2024-03-04

**Authors:** Nicholas Kim-Wah Yeo, Che Kang Lim, Katherine Nay Yaung, Nicholas Kim Huat Khoo, Thaschawee Arkachaisri, Salvatore Albani, Joo Guan Yeo

**Affiliations:** ^1^ Translational Immunology Institute, SingHealth Duke-NUS Academic Medical Centre, Singapore, Singapore; ^2^ Duke-NUS Medical School, Singapore, Singapore; ^3^ Department of Clinical Translation Research, Singapore General Hospital, Singapore, Singapore; ^4^ Rheumatology and Immunology Service, KK Women’s and Children’s Hospital, Singapore, Singapore

**Keywords:** systemic lupus erythematosus, genomics, next-generation sequencing, whole exome sequencing, monogenic, copy number variation

## Abstract

Early-onset systemic lupus erythematosus presents with a more severe disease and is associated with a greater genetic burden, especially in patients from Black, Asian or Hispanic ancestries. Next-generation sequencing techniques, notably whole exome sequencing, have been extensively used in genomic interrogation studies to identify causal disease variants that are increasingly implicated in the development of autoimmunity. This Review discusses the known casual variants of polygenic and monogenic systemic lupus erythematosus and its implications under certain genetic disparities while suggesting an age-based sequencing strategy to aid in clinical diagnostics and patient management for improved patient care.

## 1 Introduction

Systemic lupus erythematosus (SLE, or lupus) is an autoimmune disease characterized by autoantibody formation targeting nucleic components like double-stranded DNA (dsDNA) and RNA (Caielli et al., 2023). The vast spectrum of clinical manifestations ranges from mild skin rashes to widespread destructive multi-organ inflammation, which in some cases, could result in death. The pathogenesis of SLE is complex and multi-factorial (Tsokos, 2011), with genetic and environmental contributions to the disease. It has also been observed that various autoimmune diseases are more common in women (Vinuesa et al., 2023), and in SLE, individuals from Black, Asian or Hispanic ethnicities have an increased disease burden, with patients presenting with a more severe phenotype (Lewis and Jawad, 2017).

SLE can be grouped according to the age of disease onset into adult- and childhood-onset SLE (cSLE); the latter referring to those diagnosed before the age of 18 years and generally presents with greater severity especially in children under 5 years old (Bundhun et al., 2017; Alperin et al., 2018). This early onset of SLE has been associated with an increased genetic burden, highlighting the contribution of one or several risk alleles to disease (Webb et al., 2011). And within this patient group, around 3%–10% of patients carry a single disease-causing variant (Almlof et al., 2019; Belot et al., 2020; Charras et al., 2023), thus being increasingly recognized and termed as monogenic SLE (Harley and Sawalha, 2022; Vinuesa et al., 2023). Pinpointing the disease-causing variant will contribute greatly to our current knowledge of lupus pathogenesis, and this can be achieved through the use of next-generation sequencing (NGS) techniques (Sanger et al., 1977; Slatko et al., 2018; You et al., 2018; Yaung et al., 2023). As such, a focused strategy is needed together with prioritizing NGS and research efforts towards cSLE patients (Mina and Brunner, 2013).

Knowing that SLE has a strong genetic component to disease (Lewis and Jawad, 2017), multiple susceptibility loci have since been identified, following the advent of genome-wide association studies (GWAS) (Deng and Tsao, 2014). Further diving into genomic studies of SLE through NGS techniques has brought to light the utility of whole exome (WES) and whole genome sequencing (WGS). Our colleagues have also reviewed various technologies that could be employed to elucidate disease mechanisms (Yaung et al., 2023), such as Sanger sequencing (Sanger et al., 1977), single nucleotide polymorphism (SNP) array (You et al., 2018), WES and WGS (Slatko et al., 2018). In this Review, we expound further into the use of NGS techniques, notably WES, across the current genomic landscape of polygenic and monogenic SLE, discussing its potential in reconciling disease risk variants and copy number variations (CNVs) and evaluating the identification of such variants.

## 2 Next-generation sequencing in SLE

Sequencing technologies have been fundamental for researchers due to their high-throughput capabilities and more recently, their cost-effectiveness (Goodwin et al., 2016). This has allowed for comprehensive genomic studies (i.e., point mutations, small indels, CNVs) and paved the way for multi-omics studies (Levy and Myers, 2016; Lee et al., 2022; Satam et al., 2023; Yaung et al., 2023). In the context of systemic autoimmune diseases like SLE, multiple susceptibility loci identified by GWAS cumulatively contribute risk towards its development but carry a relatively low disease risk individually (Sestak et al., 2011; Wahren-Herlenius and Dorner, 2013).

Several methods have been employed in SLE genomics, including WGS, WES and targeted sequencing (Table 1). Briefly, WGS allows for comprehensive interrogation of the entire human genome and has contributed significantly to the genomic landscape via the 1000 Genomes project since 2010 (Genomes Project et al., 2010; Genomes Project et al., 2015; Sudmant et al., 2015). However, around 85% of disease-related mutations are concentrated in the exome, which constitutes about 2% of the whole genome (Majewski et al., 2011). WES then involves the selection of protein-coding regions (exons) in the genome for sequencing to identify any changes that could impact protein sequences (Ng et al., 2009). This has led to its increased use due to the significant reduction vis-à-vis the starting material, cost and data management (Petersen et al., 2017). In addition, mutations in the exonic region have been shown to be a major contributor to the development of monogenic diseases (Kuhlenbaumer et al., 2011). With the knowledge obtained from the above-mentioned methods, sequencing panels could be generated to target certain regions of interest that harbor pathogenic mutations, hence the utility of targeted sequencing for potential clinical care (Gulilat et al., 2019).

**TABLE 1:** NGS techniques and respective applications in SLE studies.

Reference(s)	Study population			Gene(s) identified	Change in sequence		Clinical manifestations (if any)
	Cases	Controls	Ethnicity		Nucleotide	Protein	
SNP array							
Han et al. (2009), Nat Genet	1,047	1,205	Han Chinese	*BLK, ETS1, IKZF1, IRF5, RASGRP3, SLC15A4, STAT4, TNFAIP3, TNFSF4, TNIP1, 6q21 7q11.23, 10q11.22, 11q23.3, 16p11.2, 22q11*	-	-	-
Rioux et al. (2009), Proc Natl Acad Sci	643	1,049	British	*HLA-DRA, TNSB-CREB1*	-	-	-
	483	746	American				
	-	672	Swedish				
Fernando et al. (2012), Ann Rheum Dis	464	468	Spanish	*BTNL2-DRA, C6orf27, DPB1-DPB2, MSH5*	-	-	-
	335	247	Filipino	*DRB1-D1A1, HLA-G-HLA-H, HLA-B-MICA, MSH5, DPB1*			
	632	742	British	*BTNL2-DRA, HLA-C-HLA-B, MUC21-PSORS1C1, TNXB-ATF6B*			
Webb et al. (2011), Ann Rheum Dis	1,569	1893	AA	*BANK1, CFB, CTLA4, C8orf13-BLK, FCGR2A, ITGAM, KIAA1542, MBL2, MECP2, MSH5, PDCD1, PTPN22, PXK, STAT4, TNFSF4*	-	-	-
	155	131	Gullah AA				
Hom et al. (2008), NEJM	1,435	3,583	European	*BLK, HLA, IRF5, ITGAM-ITGAX, STAT4*	-	-	-
	793	857	Swedish				
Gateva et al. (2009), Nat Genet	1,129	2,291	American	*ATG5, BANK1, BLK, FCGR2A, HLA-DRB1, HLA-DRB2, IRAK1-MECP2, ITGAM, IRF5, KIAA1542 (PHRF1), PTPN22, PTTG1, PXK, STAT4, TNFAIP3, TNFSF4 (OX40L), UBE2L3*	-	-	-
	834	1,338	Swedish				
Bentham et al. (2015), Nat Genet	4,036	6,959	European	*ARID5B, BANK1, BLK, CD44, CSK, CXorf21, IFIH1, IKZF1, IKZF2, IKZF3, IL10, IL12A, IRF5, IRF7, IRF8, ITGAM, JAZF1, LYST, MHC Class II, MIR146A, PCGR2A, PLD2, PTPN22, RAD51B, SH2B3, SLC15A4, SPRED2, STAT4, TNFAIP3, TNFSF4, TNIP1, TYK2, UBE2L3, UHRF1BP1, WDFY4*	-	-	-
Wang et al. (2021), Nat Commun	6,707	16,047	East Asian	*DSE, HIP1, IKZF1, NEURL4-ACAP1, PLD4, PRKCB, PRRX1-MROH9, TNFRSF13B, TYK2*	-	-	-
	4,576	8,039	European				
Elghzaly et al. (2022), Front Genet	458	769	Egyptian	*DEF6-PPARD, IRF1, IRF5, ITGAM-ITGAX, TYK2, XKR6*	-	-	-
Martínez-Bueno et al. (2018), Front Immunol	4,212	4,065	European	*HLA-DRB1, IRF7, PRDM1, SPATA8, TMEM55B*	-	-	-
Yang et al. (2015), Am J Hum Genet	1,656	3,394	East Asian	*ARID5B, CD80, CDKN1B, DRAM1, GPR19, SREBL2, TET3*	-	-	-
Yang et al. (2010), PLoS Genet	3,614	5,684	East Asian	*BANK1, BLK, ETS1, HLA-DRB1-HLA-DQA1, IRF5, STAT4, TNFAIP3, TNFSF4, WDFY4*	-	-	-
Martínez-Bueno et al. (2018), Int J Mol Sci	4,212	4,065	European	*BANK1*	-	-	-
	1761	1,138	AA				
Hanscombe et al. (2018), Hum Mol Genet	4,036	6,959	European	*HLA-DQA, HLA-DQB*	-	-	-
	1,494	5,908	AA				
WES							
Tirosh et al. (2019), Pediatr Rheumol Online J	15	-	Israeli	C1QC (premature stop codon)	c.271G>T	p.G91*	Lupus exacerbation, MAS, sepsis
				*MAN2B1*	c.192C>A	p.V56M	Dysmorphic features, decreased breath sounds bilaterally, hepatosplenomegaly, malar rash, diffuse abdominal papulosquamous rash and palmar erythema
				*SLC7A7*	c.943T>C	p.S315P	
				*PTEN*	c.697C>T	p.R233X	Macrocephaly, developmental delay, pigmented gums and macules of the glans penis; previously reported by Liaw et al., 1997, Nat Genet.
				STAT1 gain-of-function	c.862A>G	p.T288A	Chronic mucocutaneous candidiasis and autoimmunity
Batu et al. (2018), J Rheumatol	7	245	Turkish	*C1QA*	c.622C>T	p.Q208*	-
				*C1QC*	c.79C>T	p.Q27*	
					c.100G>A	p.G34R	
				*C1S*	c.1945G>C	p.A649P	
				*DNASE1L3*	c.289_290delAC	p.T97lefs*2	
				*HDAC7*	c.163C>T	p.R55W	
Brown et al. (2022), Nature	1	-	European	*TLR7*	c.790T>C	p.Y264H	Inflammatory arthralgias, constitutional symptoms, intermittent hemichorea episodes with hypertensive crisis
	1	-	American		c.82A>G	p.R28G	Neuromyelitis optica in the presence of ANAs and antibodies to aquaporin-4
	1	-	East Asian		c.1521T>G	p.F507L	Malar rash, join pain, Raynaud’s phenomenon, alopecia, fever, oral ulcers
Lee et al. (2022), Pediatr Rheumatol Online J	184	-	East Asian	*CFHR4*	c.103T>C	-	FTT, fever, arthritis, discoid rashes
				*C1S*	c.1241G>A	p.R414H	Oral ulcerations, swelling legs, seizure with posterior reversible encephalopathy syndrome
				*C2*	c.1558C>T	p.R520C	
				*DNASE1*	c.370G>A	p.E124K	
				*DNASE1L3*	c.764G>A	p.R225K	Nephrotic-range proteinuria, malar rash, oral ulceration, arthritis
				*SLC7A7*	c.625 + 1 G>A	-	Twin 1: transient proteinuria, lymphopenia, thrombocytopenia, low C4, positive auto-Ab profile Twin 2: FTT, glomerulonephritis with profound proteinuria
				*TREX1*	c.292_293 ins A	p.C99M fs	FTT, chilblain-like skin lesions, dystonic posturing with peripheral spasticity
Delgado-Vega et al. (2018), Sci Rep	5	-	Icelandic	*ANKRD50*	-	p.T367M	-
				*CHD3*		p.A1523T	
				*CLC*		p.N65K	
				*DCLRE1C*		p.H283N	
				*FAM71E1*		p.L7F	
				*FAM8A1*		p.G234R	
				*FAT4*		p.P247T	
				*FBXL14*		p.N102H	
				*KIR2DS4*		p.I255L	
				*KRTAP4-9*		p.D18V	
				*MPHOSPH8*		p.E499K	
				*NOTCH1*		p.D932N	
				*NUP214*		p.I765V	
				*PABPC3*		p.A114T	
				*PDHA2*		p.R286P	
				*SCL25A9*		p.G103R	
				*TPRA1*		p.E300K	
				*WDR25*		p.R206H	
				*XRCCBP1*		p.A229V	
Hong et al. (2022), Front Pediatr	1	-	East Asian	*ACP5*	c.420G>A	p.R46Q	Recurrent upper respiratory tract infections and oral thrush throughout life; presented with cutaneous bleeding spots on lower extremities
					c.1152G>T	p.G290V	
				*SAMDH1*	c.1423G>A	p.R408H	
Li et al. (2020), Medicine (Baltimore)	7	-	East Asian	*NRAS*	c.38A>G	p.G13C	Genetic changes observed in Patients 1-4
				*PIK3CD*	c.3061G>A	p.E1021K	Genetic changes observed in Patients 6 and 7
				*TNFAIP3*	c.559C>T	p.Q187X	
Demirkaya et al. (2017), Arthritis Rheumatol	4	5	Turkish	*C1R*	c.1332delT	p.P445L fs*11	Systemic inflammation, presence of ANA, malar rash
Tang and Luo. (2022), Arch Rheumatol	4	-	East Asian	*CR2*	C.2804T>C	p.I935T	-
				*ITGB3*	c.1960G>A	-	
Raupov et al. (2022), Front Pediatr	2	-	Russian	*RNASEL*	c.1880A>G	p.K627R	Twin 1: petechiae, fever, Henoch-Schonlein purpura Twin 2: acute severe leg pain, petechiae
WGS							
Almlof et al. (2019), Hum Genet	71	2,711	Swedish	*C1QC*	C>T	p.R69*	-
				*C1S*	G>A	p.D631N	
				*DNASE1*	G>A	p.G127R	
					C>G	p.P154A	
				*DNASE1L3*	G>A	p.T224M	
				*IFIH1*	G>A	p.R77W	
					G>A	p.R374C	
				*RNASEH2A*	A>G	p.K221R	
Almlof et al. (2021), Eur J Hum Genet	71	2,711	Swedish	*ISX*	c.1076G>A	p.R138N	-
				*LTB4R2*	c.620C>T	p.N169*	
				*MAZ*	c.1276T>G	p.C368G	
				*PPARA*	Deletion 80 kb from TSS in intron 6		
				*RBM10*	Deletion of exons 3-7		
				*SMARCA2*	Deletion 603 bases downstream		
Targeted Sequencing							
Alghamdi et al. (2021), Gene	100	147	Egyptian	*AIRE*	AIRE (rs2075876) variant conferred protection against developing SLE, but not the CTLA4 (rs231775) variant		
				*CTLA-4*			
Lundtoft et al. (2022), Arthritis Rheumatol	2,290	1,251	Scandinavian	*C4*	Low copy number associated with increased risk for SLE (stronger for C4A than C4B)		
Montufar-Robles et al. (2019), Cell Immunol	379	460	Mexican	*AIRE*	Ser196Ser synonymous variant associated with SLE		
Mueller et al. (2013), Am J Hum Genet	4	-	West African	*FCGR3B*	Reduced copy number associated with SLE		
	3		East Asian				
	2		CEPH				
Raj et al. (2020), Genome Biol	1700	2,108	Caucasian	*DAP1*	Risk haplotype downregulates transcription, leading to increased autophagy, diminished apoptosis, increased humoral autoimmunity, increased risk for discoid rash		
Sandling et al. (2021), Ann Rheum Dis	958	1,026	Swedish	*ARRDC5*	-	p.N231R	-
				*BNC2*		p.H307Y	
				*C1orf27*		p.N246K	
				*CAD*		p.M922K	
				*CAMK2G*		p.H370Q	
				*CCDC141*		p.S1522F	
				*DAD1*		p.S66R	
				*IFNA21*		p.Y146C	
				*IQGAP1*		p.V1371M	
				*KIR3DL3*		p.G219D	
				*MUC5B*		p.T2727P	
				*PIK3R2*		p.G372S	
				*PMEL*		p.P402H	
				*RUNDC1*		p.V477I	
				*SCNN1A*		p.R511*	
				*SELENBP1*		p.P36T	
				*SENP1*		p.T155A	
				*TMEM132C*		p.P1094A	

AA, African-American; ANA, anti-nuclear antibody; CEPH: Centre d'Etude du Polymorphisme Humain; del, deletion; fs, frameshift; FTT, failure-to-thrive; ins, insertion; MAS, macrophage activation syndrome; NGS, next generation sequencing; SLE, systemic lupus erythematosus; SNP, single nucleotide polymorphism; WES, whole exome sequencing; WGS, whole genome sequencing. Italic values denote genes.

### 2.1 Polygenic contribution to SLE

Autoimmune diseases have been known to arise from an accumulation of genetic and environmental factors across one’s lifetime, as in the case of adult-onset SLE (Goodnow et al., 2005). More than 100 loci associated with SLE have been identified through GWAS (Wang et al., 2021), such as regions in the Human Leukocyte Antigen (*HLA*) locus (Hanscombe et al., 2018), *STAT4* (Remmers et al., 2007; Han et al., 2009), *TNFSF4* (Han et al., 2009), *BANK1* (Kozyrev et al., 2008; Martinez-Bueno et al., 2018), *TNFAIP3* (Graham et al., 2008; Musone et al., 2008; Han et al., 2009), *BLK* (Hom et al., 2008; Han et al., 2009), *IRF5* (Jones et al., 2019), *ETS1* (Han et al., 2009; Yang et al., 2010; Jones et al., 2019), *WDFY4* (Yang et al., 2010) and *TNIP1* (Han et al., 2009; Yang et al., 2010; Jones et al., 2019). However, these variants are unlikely to contribute significantly to SLE pathogenesis individually, unless coupled either with variants in certain regulatory regions or in other genes that maintain immune tolerance (Jones et al., 2019). Importantly, epistatic interaction between genes may contribute in part to the development of complex diseases such as lupus (Hughes et al., 2012; Wei et al., 2014).

It has been recently suggested that polygenic risk scores (PRS) could be utilized to identify and stratify potential SLE patients for early intervention, if needed (Khunsriraksakul et al., 2023). Briefly, GWAS-identified risk variants are statistically compiled to predict disease incidence in a population and risk for developing SLE in individuals (Khunsriraksakul et al., 2022). An association between a high PRS and poorer prognosis in SLE has been observed (Chen et al., 2020; Reid et al., 2020; Sandling et al., 2021), with one study going further to delineate T cell differentiation and innate immunity as the two key axes of SLE association mediated by HLA and interferons (IFNs) respectively (Sandling et al., 2021). The strong involvement of HLA and IFNs has also been described for SLE pathogenesis (Chen et al., 2017; Villarino et al., 2017; Alunno et al., 2019; Crow and Ronnblom, 2019). Despite its utility, PRS has yet to be generalizable beyond the specific population being studied, which further emphasizes the need for larger, diverse and well-represented datasets in order to draw meaningful conclusions (Torkamani et al., 2018). In addition, data generated from GWAS is primarily based on SNP arrays which can be limited by its inability to identify causal variants and ultra-rare mutations, particularly in ethically under-represented populations (Tam et al., 2019). NGS techniques thus provide an answer to interrogating such variants, which might aid in enriching our knowledge of SLE pathogenesis, the clinical diagnosis and management of polygenic SLE together with the potential use of PRS.

### 2.2 Monogenic contribution to SLE

Single gene defects are part of the diverse heterogenous etiologies for lupus, where about 1%–3% of SLE patients carry a single mutation that leads to disease development (Costa-Reis and Sullivan, 2017). Albeit rare, monogenic SLE is characterized by a more severe phenotype early in life (Webb et al., 2011; Vinuesa et al., 2023). Several gene sets involved in the complement pathway, IFN responses, nucleic acid sensing and immune tolerance have been implicated in the pathogenesis of monogenic SLE (Alperin et al., 2018; Vinuesa et al., 2023).

Most complement-related SLE defects are found in the C1 (*C1QA*, *C1QB*, *C1QC*, *C1R*, *C1S)* (Lood et al., 2009; Bienaime et al., 2010; Demirkaya et al., 2017; Almlof et al., 2019) or C4 (*C4A*, *C4B*) compartments (Blanchong et al., 2001; Vinuesa et al., 2023). Other affected regions include the *C2* and *C3* genes (Miller and Atkinson, 2012; Vinuesa et al., 2023). Complement-deficient patients tend to have an impaired clearance of cellular apoptotic fragments, which in turn facilitates autoimmunity (Costa-Reis and Sullivan, 2017; Vinuesa et al., 2023). Next, studies have observed an elevated IFN signature in SLE patients (Baechler et al., 2003; Reynier et al., 2011), and the association of variants in *ADAR1* (Crow and Ronnblom, 2019), *TREX1* (Rice et al., 2015), *SAMHD1* (Abdel-Salam et al., 2010; Ravenscroft et al., 2011) and *IFIH1* (Rice et al., 2014; Almlof et al., 2019) genes to disease (Abdel-Salam et al., 2010; Ravenscroft et al., 2011; Rice et al., 2012; Rice et al., 2014; Crow et al., 2015). *ADAR1*, *TREX1* and *SAMHD1* are involved in nucleic acid metabolism while *IFIH1* is involved in nucleic acid sensing. Lastly, defects in nucleic acid sensing and degradation genes *DNASE1* (Yasutomo et al., 2001; Almlof et al., 2019), *DNASE1L3* (Al-Mayouf et al., 2011; Almlof et al., 2019) and *TLR7* (Giltiay et al., 2013; Brown et al., 2022) have been found in SLE patients. This causes an accumulation of extracellular nucleic acids, leading to TLR7 activation and downstream type I IFN production. Type I IFN further upregulates TLR7 expression, creating a positive feedback loop that eventuates into autoantibody production (Caielli et al., 2023). With this, it has been recently found that two *TLR7* variants (Y264H and F507L) were recently identified to cause SLE, with the Y264H variant presenting with an increased sensing of guanosine and 2′,3′-cGMP (Shibata et al., 2016; Zhang et al., 2016; Zhang et al., 2018; Brown et al., 2022).

Across autoimmune diseases, a hallmark of its development is the loss of tolerance to self-antigens, with *AIRE* and *CTLA-4* being implicated in SLE (Pullmann et al., 1999; Ahmed et al., 2001; Hudson et al., 2002; Lee et al., 2005; Cunninghame Graham et al., 2006; Lovewell et al., 2015; Montufar-Robles et al., 2019; Alghamdi et al., 2021). *AIRE*, or autoimmune regulator is essential for maintaining central immune tolerance by controlling the negative thymic selection of hyper-reactive T lymphocytes against self-antigens (Yang et al., 2015). Mutations in this gene region have been observed in Norwegian patients with autoimmune polyendocrine syndrome type I (APS-1; (Oftedal et al., 2023)) and Japanese patients with rheumatoid arthritis (RA; (Terao et al., 2011)). More recently, an *AIRE* Ser196Ser synonymous variant was associated with SLE in a recent targeted sequencing study in a Mexican cohort (Montufar-Robles et al., 2019). However, when extended to GWAS performed on a larger European SLE cohort, no association was found (Bentham et al., 2015). Next, cytotoxic T-lymphocyte associated protein 4 (CTLA-4, or CD152) is an important checkpoint inhibitor in peripheral immune tolerance via negative signaling in regulating autoreactive T cells (Liu and Zhang, 2013; Van Coillie et al., 2020). Though several reports have identified certain polymorphisms contributing to SLE development (Pullmann et al., 1999; Ahmed et al., 2001; Hudson et al., 2002; Lee et al., 2005; Cunninghame Graham et al., 2006; Jury et al., 2010), a meta-analysis has highlighted no association of said variants to lupus (Liu and Zhang, 2013; Alghamdi et al., 2021). In some cases, specific *CTLA-4* variants could even contribute to protection against SLE (Barreto et al., 2004), suggesting that only certain variants within the *CTLA-4* gene region have an association with SLE development.

Recent studies have described several novel genes associated with SLE following WES analysis in an Asian population, such as the decreased expression of cell division cycle 27 (CDC27) in patients (Shang et al., 2022), and novel variants in genes encoding for complement receptor 2 (CR2) (Tang and Luo, 2022), C1R (Demirkaya et al., 2017), NRAS, TNFAIP3 and PIK3CD (Li et al., 2020), WNT16 and ERVW-1 (Chen et al., 2022), ACP5 and SAMHD1 (Hong et al., 2022). This list of genes contributing to monogenic SLE continues to grow with increased usage of WES over the past few years, further enriching our knowledge about the genetic contribution to SLE.

### 2.3 Copy number variation (CNV)

Copy number variation (CNV) is a phenomenon where repeated genomic sequences occur and arise from the process of genomic rearrangement, which can manifest as translocations, inversions, insertions and deletions (Feuk et al., 2006; Human Genome Structural Variation Working et al., 2007). However, the total number of gene copies and its downstream effects may vary between individuals (Usher and McCarroll, 2015). In the past two decades, several CNVs associated with SLE development have been identified, such as C4 (*C4A*, *C4B*) (Yang et al., 2007; Pereira et al., 2019; Kamitaki et al., 2020; Lundtoft et al., 2022), *FCGR3A*, *FCGR3B* (Willcocks et al., 2008; Niederer et al., 2010), *CCL3L1* (Gonzalez et al., 2005), *RABGAP1L* (Kim et al., 2013), *TLR7* (Garcia-Ortiz et al., 2010) and *HSP90* (Zhang et al., 2019).

As previously mentioned, defects in complement genes have been observed to be a monogenic cause of SLE. Of note, C4, or complement compartment protein 4, is usually present in most individuals as two copies of *C4A* and *C4B* respectively. In some cases, SLE patients may carry a range of zero to five copies of *C4A* and zero to four copies of *C4B* (Yang et al., 2007; Pereira et al., 2019). A recent study has described an association between a low C4A copy number and an increased risk of developing SLE (Kamitaki et al., 2020). Though C4 genes are highly homologous and are usually excluded from variant calling analysis, Lundtoft *et al.* performed a focused analysis into C4 CNVs via targeted sequencing and found Scandinavian SLE patients with a low *C4A* copy number and carrying a common loss-of-function (LoF) variant presenting with lowered plasma C4 levels (Lundtoft et al., 2022). Whether this phenomenon can be extended to other ancestral populations remains unknown and warrants further investigation.

Other genes like *FCG3RA* and *FCGR3B* encode for low-affinity Fc gamma (Fcγ) receptors of IgG and are crucial in the binding and clearing of immune complexes (Willcocks et al., 2008; Niederer et al., 2010), while *CCL3L1* (C-C chemokine ligand 3 like-1) translates into a ligand that binds to C-C chemokine receptor 5 (CCR5) (Gonzalez et al., 2005). Healthy individuals carry two copies of each respective gene, but SLE risk increases when there are either lower or higher copy numbers of said genes (Willcocks et al., 2008). Increased SLE susceptibility was also observed with low *RASGAP1L* and high *TLR7* copy numbers respectively. *RASGAP1L* encodes for a Rab GTPase-activating protein (Kim et al., 2013), while *TLR7* is a key receptor in innate immunity that recognizes single-stranded RNA (Lund et al., 2004; Takeda and Akira, 2005). Lastly, abnormal CNVs in heat shock proteins 90 (HSP90), especially in its AB1 isoform, were identified to correlate with SLE in the Han Chinese (Zhang et al., 2019). This highlights the importance of CNVs in SLE and autoimmunity and thus the need for more traction toward implementing a pipeline to include them in future genetic screens (Zhao et al., 2020).

### 2.4 Identification of potential disease-causing variants

Genetic testing using NGS techniques has identified potential disease-causing variants and led to better preventative risk management of diseases (Shaw et al., 2023). However, given its complexity, the labeling of variants as potentially pathogenic should be done with caution to prevent misdiagnoses. A misdiagnosis of a pathogenic variant can result in unnecessary medical interventions and cause undue psychological distress to both patients and their families (Manrai et al., 2016; Shaw et al., 2023). Such detrimental consequences have occurred in diseases like hypertrophic cardiomyopathy and cancers, where variants that were thought to be pathogenic were subsequently found to be benign due to the under-representation of certain ancestries in reference control groups (Manrai et al., 2016; Shaw et al., 2023).

To prevent such genetic misclassifications, the American College of Medical Genetics and Genomics has introduced a standardized framework for variant interpretation (Richards et al., 2015). In the case of SLE and other autoimmune diseases, pathogenic variants can be better identified prior to further functional validation through this framework, thus reducing the occurrence of false positives as the number of sequencing studies continues to rise (Vinuesa et al., 2023). In addition, various consortia like the Clinical Genome Resource (ClinGen; (Rehm et al., 2015)), Rheumatologic Autoimmune Clinical Domain Working Group under ClinGen, Lupus in Minority Populations, Nature *versus* Nurture (LUMINA; (Alarcon et al., 2001)) have been established to aggregate all available genomic data and concentrate global research efforts. Crucially, the consolidation of genomic data overcomes the major limitation of genome-wide studies of requiring large sample sizes due to the need to adopt a high level of significance to account for multiple testing (Tam et al., 2019). With this framework for variant interpretation and genomic data from various consortia, this can be potentially applied to the dysmorphic syndromes associated with SLE, specifically genes of the Ras/mitogen-activated protein kinase (Ras/MAPK) pathway to identify with greater certainty the potential pathogenic genetic variants within this pathway that contribute to SLE (Amoroso et al., 2003; Lisbona et al., 2009; Leventopoulos et al., 2010; Hanaya et al., 2017; Uehara et al., 2018). However, further investigations would be needed to delineate the underlying mechanism with functional studies of the different genes in the Ras/MAPK pathway as these are currently described in case reports and series.

Our current understanding has informed us that certain ancestral groups have an increased predilection towards developing SLE (Lewis and Jawad, 2017), which requires controlling for in future sequencing studies to prevent any potential misclassification of disease-causing variants due to the unavailability of an adequate ancestry-specific reference genome. Past research has been largely focused on European ancestry (Yang et al., 2007; Lewis and Jawad, 2017; Hanscombe et al., 2018), resulting in an under-representation of data from other ancestries to draw meaningful generalizations about the disease. This can be resolved by tapping on several biobanks that have been consolidated over the years to provide greater depth and insights into the genetic differences within and across various ancestries. These include, and are not limited to, the Tohoku Biobank (150,000 participants; (Minegishi et al., 2019)), Mexican Biobank (6,057 participants; (Sohail et al., 2023)), Biobank Japan (BBJ, 260,000 participants; (Kanai et al., 2018)), China Kadoorie Biobank (500,000 participants; (Chen et al., 2011)), H3Africa (70,000 participants; (Consortium et al., 2014; Mulder et al., 2018)), UK Biobank (500,000 participants; (Bycroft et al., 2018; Van Hout et al., 2020; Gaynor et al., 2023)), Michigan Genomic Initiative (MGI, 91,000 participants; (Zawistowski et al., 2023)), Vanderbilt University Biobank (BioVU, 300,000 participants; (Khunsriraksakul et al., 2023)), and SG10K (9,051 participants; (Chan et al., 2022)). It should be noted that SG10K has since been expanded to SG100K, whereby data from 70,000 participants across four national cohort studies will be pooled together with the additional recruitment of 30,000 individuals (Begum, 2022).

## 3 Discussion

In this Review, we have provided an overview of various susceptibility genes contributing to the development of SLE either through a polygenic or monogenic route identified via NGS techniques, highlighted the involvement and importance of CNVs and urged for the inclusiveness of control groups to account for ancestral differences to prevent any potential variant misclassification.

The introduction of WGS and WES has resulted in faster genomic interrogation, allowing for one’s entire genome to be generated in a matter of days to weeks (Bourchany et al., 2017; Duncavage et al., 2021). The data generated from WGS provides comprehensive information on both intronic (non-coding) and exonic (protein-coding) regions. However, the contributions of non-coding variants towards disease have yet to be thoroughly elucidated and the downstream analyses of such intronic regions remain complex and highly challenging (Zhao et al., 2020). As such, WES has become increasingly popular in clinical diagnostics and research due to its utility (∼95% capture of exonic and splice site regions (Field, 2021; Zhang et al., 2021)), ease of analysis (Yaung et al., 2023) and lower cost (one-third that of WGS (Goodwin et al., 2016; Field, 2021))

In addition, structural variants like CNV are relatively common across the whole genome (with a frequency of around 12%; (Iafrate et al., 2004; Sebat et al., 2004; Tuzun et al., 2005; Conrad et al., 2006; McCarroll et al., 2006)), and can influence gene expression (Somerville et al., 2005; Lee et al., 2006; McCarroll et al., 2006). As we have alluded to the growing importance of CNV in SLE immunogenetics, the coupling of WES with CNV detection addresses the need for a holistic interrogation of the genetic contribution to SLE through the dual identification of variations in exonic sequences and gene copy numbers. This is achievable with tools such as CoNIFER (Krumm et al., 2012), exomeCopy (Love et al., 2011), CNVkit (Talevich et al., 2016), cn. MOPS (Klambauer et al., 2012), CNest (Fitzgerald and Birney, 2022), CNVind (Kusmirek and Nowak, 2022), CoverageMaster (Rapti et al., 2022) and EXCAVATOR2 (D’Aurizio et al., 2016; D’Aurizio et al., 2018). More recently, Olfe *et al.* have demonstrated CTLA-4 insufficiency due to a novel *CTLA-4* deletion using ClinCNV (German and Stephan, 2019; Olfe et al., 2023), further highlighting the synergy of CNV calling with WES analysis. Beyond the scope of autoimmune diseases, NGS techniques have also been extensively utilized in identifying causal variants (including CNVs) contributing to cancer (van Dijk et al., 2014; Papp et al., 2021; Satam et al., 2023), congenital (Lai et al., 2021; Li et al., 2022; Liu et al., 2022; Wang et al., 2022; Wu et al., 2022; Refeat et al., 2023), cardiovascular (Hu et al., 2023) and hematological diseases (Hassan et al., 2023).

Though the method of WES has been well-established over the years, notable limitations persist in WES-based CNV analyses. The technique primarily targets coding regions, leading to a restricted view of the genome and potentially missing important regulatory components within non-coding regions such as intergenic or intronic regions (Mandelker et al., 2016; Royer-Bertrand et al., 2021). This significantly impacts the sensitivity of CNV detection. In addition, it is susceptible to biases, such as GC content bias, which can impact the reliability of CNV calls (Lelieveld et al., 2015). Furthermore, a relatively higher false positive rate and the limitation of achieving homogeneous coverage of sequencing reads restrict its inclusion as a gold-standard method for CNV detection (Marchuk et al., 2018; Burdick et al., 2020). These limitations emphasize the necessity of integrating WES with other omics approaches for more accuracy in CNV detection (Gabrielaite et al., 2021). Nonetheless, with ongoing upgrades to sequencing libraries, capture kits and bioinformatics pipelines, it is anticipated that the existing limitations will be alleviated (Zhou et al., 2021). Future applications of third-generation sequencing (TGS) techniques such as long-read sequencing hold promise in addressing these constraints and provide additional possibilities in detecting structural variations (SVs) (Xiao and Zhou, 2020).

Though SLE is known to have a strong genetic predilection, its typical development is usually due to polygenic contributions coupled with an environmental trigger (Harley and Sawalha, 2022); the latter of which must not be ignored. Research into the host-environment interplay has yielded physical/chemical factors (smoking, chemical exposure (Kilburn and Warshaw, 1992; Speyer and Costenbader, 2018; Akhil et al., 2023)), Epstein-Barr virus (EBV) infections (Poole et al., 2006; Jog and James, 2020), gut microbiota (Neuman and Koren, 2017) and obesity (Kang et al., 2020) as contributors to the development of SLE (Parks et al., 2017; Gulati and Brunner, 2018; Akhil et al., 2023). Such environmental triggers can influence methylation patterns in genes related to B and T cells, which are associated with SLE pathology (Akhil et al., 2023). These include observations of hypomethylation in *CD40L* (Vordenbaumen et al., 2021) and *CD70* (Keshavarz-Fathi et al., 2022), as well as hypermethylation of *FOXP3* (Hanaei et al., 2020) and *CTLA-4* (Nosrat zehi et al., 2021).

Elucidating the pathogenesis of autoimmune diseases like SLE remains complex, and studies have called for the need for a multi-omics approach to furnish our current understanding of the disease (Fang et al., 2016; Hedrich, 2017; Kwon et al., 2019; Yaung et al., 2023). Thus far, transcriptomic signatures obtained from blood and tissues have shown an enrichment of genes involved in the IFN response (Banchereau et al., 2016; Der et al., 2019), which corroborates with previous genetic data (Baechler et al., 2003; Reynier et al., 2011). Epigenetic modifications in the genome such as methylation (Ballestar, 2011; Hedrich, 2017), non-coding RNAs (Taheri et al., 2020) and post-translational histone modifications (i.e., methylation, acetylation; (Hu et al., 2008)) have also been associated with the development of SLE. Proteomic studies have proven difficult to isolate biomarkers for diagnosis, management and monitoring due to the heterogeneity of the disease and its involvement across multiple organs (ref), but current efforts continue to show some promise (Huang et al., 2022; Fasano et al., 2023). Indeed, more needs to be done to reconcile multi-omics and genetic data of SLE in the future.

## 4 Conclusion

Up to 10% of patients below the age of 18 years can carry a significant disease-causing variant which manifests as severe SLE, alluding to a monogenic etiology and highlights the value of doing NGS in children with a very early onset of disease (Alperin et al., 2018; Charras et al., 2021). Previous studies have shown the utility of WES in unraveling novel rare variants and determining its respective contribution(s) to disease (Pullabhatla et al., 2018; Almlof et al., 2019; Tirosh et al., 2019; Almlof et al., 2021). However, genetic variation across ancestries should not be overlooked to prevent variant misclassification and downstream misdiagnoses. This can be controlled via the inclusion of gene datasets across various biobanks, consortia and databases. With that, establishing a pipeline where WES and CNV detection are coupled together will allow for the timely and pinpoint clinical diagnosis of SLE to allow for better clinical management and intervention.

## 5 Search strategy and selection criteria

We searched PubMed between 30 August 2023 and 7 February 2024, using the terms “systemic lupus erythematosus (SLE)”, “next-generation sequencing (NGS)”, “genomics”, “copy number variation” in articles published from 1 Jan 2013 until 7 February 2024. Articles were also identified through references from articles identified through the search. Only papers published in English were reviewed and the final reference list was generated based on the relevance to the scope of this Review.
